# Fungal esophagitis associated with tuberculous pericarditis in an human immunodeficiency virus-positive patient: a case report

**DOI:** 10.1186/s13256-022-03561-x

**Published:** 2022-11-07

**Authors:** Gleiciere Maia Silva, Bruna Rodrigues de Sousa, Kaliny Benicio Torres, Rejane Pereira Neves, Heloisa Ramos Lacerda de Melo, Reginaldo Gonçalves de Lima-Neto

**Affiliations:** 1 Post-Graduate Program in Tropical Medicine, Hospital of Clinics–Bl, Hospital das Clínicas of the Federal University of Pernambuco (UFPE), Av. Prof. Moraes Rego, s/n, Cidade Universitaria, Recife, PE 50670-901 Brazil; 2 Post-Graduate Program in Fungal Biology, Center for Biosciences, Federal University of Pernambuco, Av. da Engenharia, s/n, Cidade Universitaria, Recife, PE 50670-901 Brazil; 3 Realab, Real Hospital Português, Av Agamenon Magalhães, 4760, Paissandu, Recife, PE 52010-075 Brazil; 4 Department of Tropical Medicine, Center for Medical Sciences, Federal University of Pernambuco, Av. da Engenharia 531-611, Recife, 50670-901 Brazil

**Keywords:** Fungal infection, Yeast, Tuberculous pericarditis, PLHIV, Case report

## Abstract

**Background:**

Opportunistic infections are frequent in people living with the human immunodeficiency virus who either do not have access to antiretroviral therapy (ART) or use it irregularly. Tuberculosis is the most frequent infectious disease in PLHIV and can predispose patients to severe fungal infections with dire consequences.

**Case presentation:**

We describe the case of a 35-year-old Brazilian man living with human immunodeficiency virus (HIV) for 10 years. He reported no adherence to ART and a history of histoplasmosis with hospitalization for 1 month in a public hospital in Natal, Brazil. The diagnosis was disseminated *Mycobacterium tuberculosis* infection. He was transferred to the health service in Recife, Brazil, with a worsening condition characterized by daily fevers, dyspnea, pain in the upper and lower limbs, cough, dysphagia, and painful oral lesions suggestive of candidiasis. Lymphocytopenia and high viral loads were found. After screening for infections, the patient was diagnosed with tuberculous pericarditis and esophageal candidiasis caused by *Candida tropicalis*. The isolated yeasts were identified using the VITEK 2 automated system and matrix-assisted laser desorption/ionization time-of-flight–mass spectrometry. Antifungal microdilution broth tests showed sensitivity to fluconazole, voriconazole, anidulafungin, caspofungin, micafungin, and amphotericin B, with resistance to fluconazole and voriconazole. The patient was treated with COXCIP-4 and amphotericin deoxycholate. At 12 days after admission, the patient developed sepsis of a pulmonary focus with worsening of his respiratory status. Combined therapy with meropenem, vancomycin, and itraconazole was started, with fever recurrence, and he changed to ART and tuberculostatic therapy. The patient remained clinically stable and was discharged with clinical improvement after 30 days of hospitalization.

**Conclusion:**

Fungal infections should be considered in patients with acquired immunodeficiency syndrome as they contribute to worsening health status. When mycoses are diagnosed early and treated with the appropriate drugs, favorable therapeutic outcomes can be achieved.

## Background

Fungi are opportunistic microorganisms that contribute to poor outcomes in patients with advanced human immunodeficiency virus (HIV) infections. These virus-associated infections are among the world’s major public health problems [1, 2]. According to the Joint United Nations Programme on HIV/AIDS (UNAIDS), more than 37.7 million people worldwide live with HIV, of whom 28.2 million have access to antiretroviral therapy (ART). Nevertheless, since the beginning of the HIV epidemic, 36.3 million people have died from illnesses related to acquired immunodeficiency syndrome (AIDS) [1].

Several yeast species affect people living with the human immunodeficiency virus (PLHIV), among which *Candida* species are the most common. The spectrum of clinical manifestations varies from the asymptomatic to severe forms. *Candida* causes various clinical infections, including oropharyngeal candidiasis, esophageal candidiasis, cutaneous candidiasis and candidemia [3]. *Candida* species are associated with oral and esophageal infections, and most respond efficiently to antifungals. However, levels of antifungal resistance (especially to azoles by non-albicans *Candida* species) have been increasing [2, 4, 5]. In addition, non-albicans *Candida* species, such as *C. tropicalis*, *C. krusei*, and *C. glabrata* (which exhibit reduced intrinsic sensitivity to azole antifungals), are isolated at high frequency among PLHIV [3].

Likewise, HIV infection is a primary risk factor for tuberculosis (TB) [6, 7], and individuals living with HIV/AIDS are 21- to 34-fold more likely to develop active TB and its complications than the general population [8, 9]. Recently, a high prevalence of tuberculous pericarditis in AIDS patients in Brazil has been reported [10].

We present the case report of a patient with AIDS who was non-compliant with ART and developed fungal complications associated with disseminated TB. Nevertheless, he achieved a favorable outcome. Due to the increased number of disseminated TB cases associated with fungal infections in Brazil, uncommon occurrences should be highlighted and reported to avoid delayed diagnoses.

## Case presentation

A 35-year-old Brazilian man with HIV for 10 years who was non-adherent to ART (lamivudine + abacavir + dolutegravir) was admitted with a history of successfully treated histoplasmosis. He had been hospitalized for 1 month at a public hospital in Natal, Brazil, with initial symptoms of intense diarrhea, daily fevers, sweating, and chills for 3 weeks. He was diagnosed with disseminated TB, and tuberculostatic treatment (isoniazid, rifampin, ethambutol, streptomycin, and pyrazinamide) was given for 10 days. However, due to increased transaminase levels, his regimen was changed to levofloxacin, capreomycin, and ethambutol. He was observed for 10 days and discharged.

Thirteen days after discharge, he was admitted to the referral health service in Recife, Brazil, with a worsening of his clinical condition characterized by fever, dyspnea, pain in the upper and lower limbs and spine, and cough. Prophylactic treatment with sulfamethoxazole + trimethoprim + piperacillin/tazobactam was initiated.

**Fig. 1:**
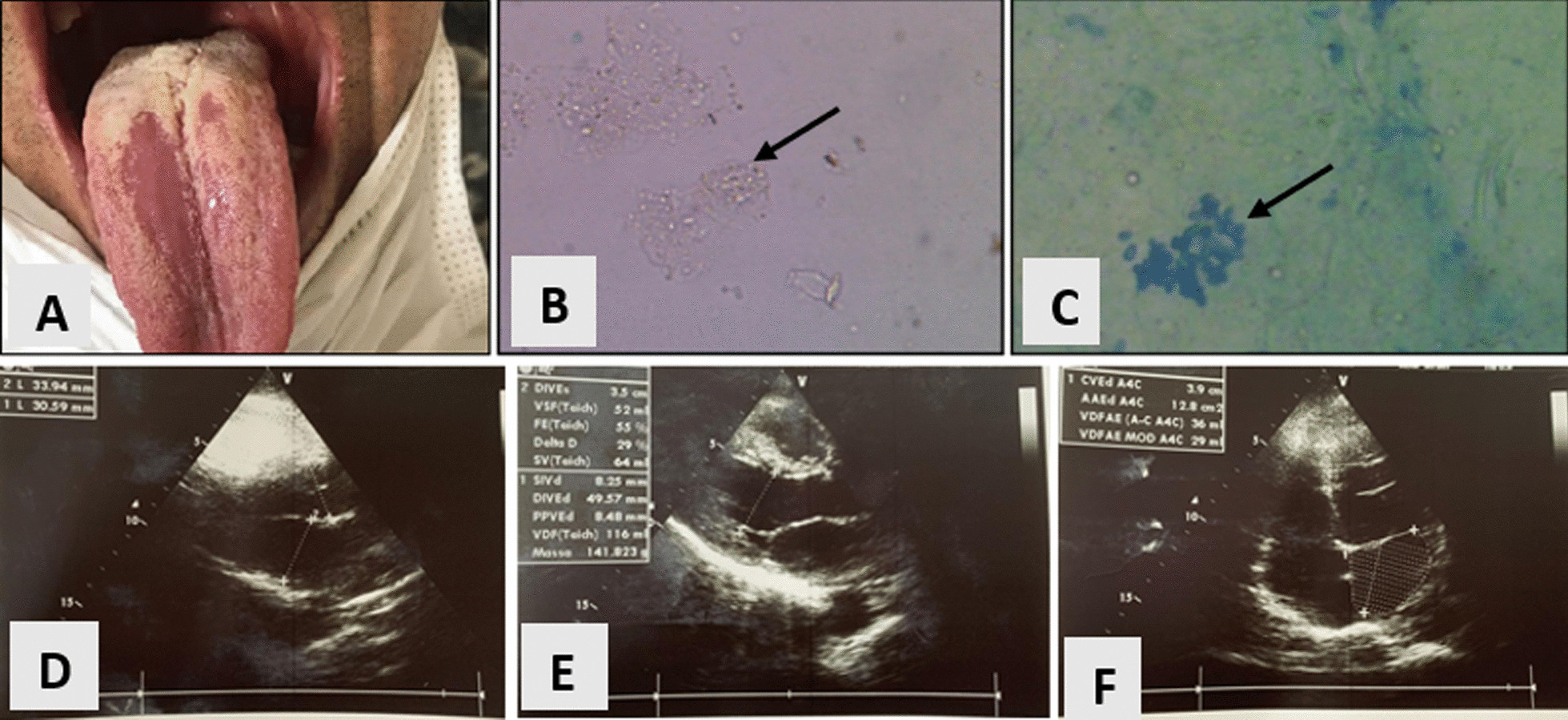
**a** White-yellowish plaques on the tongue. **b**,** c** Direct mycological examination of the oral secretion showing hyaline yeast cells without dye (**b**) stained with methylene blue (**c**) (magnification: ×400). **d**–**f** Thickened pericardium with areas of hyperechogenicity in the left ventricular wall and absence of pericardial effusion

Lymphocyte counts showed that patient was severely immunocompromised, with a CD4 count of 8 cells/mL, CD8 count of 347 cells/mL, and CD4/CD8 ratio of 0.02. A high viral load was detected (174,034 copies). Several laboratory tests were requested, including fungal investigations because of his history of histoplasmosis 2 years prior and the finding of several whitish plaques in his oral cavity (Fig. 1a). Blood culture, bronchoalveolar lavage, and oral secretions were obtained. The samples were sent to the Laboratory of Diagnostic in Tropical Diseases at the Federal University of Pernambuco, and slides were made for direct examination (Fig. 1b, c) to visualize fungal structures. Spherical yeast cells were observed in large numbers.

Each biological sample was cultured on Sabouraud Dextrose Agar (Difco, Thermo Fisher Scientific, Waltham, MA, USA) + chloramphenicol in duplicate, and the plates were maintained at 28 °C and 37 °C. There was rapid growth with sporulation after 3 days of culture. Yeasts were identified using the VITEK® 2 automated microbial identification system (bioMérieux SA, Marcy-l'Étoile, France) and matrix-assisted laser desorption/ionization time-of-flight–mass spectrometry according to Lima-Neto *et al*. [11]. Both methods identified *Candida tropicalis*.* In vitro* antifungal susceptibility tests were performed according to the protocol described in the 2008 Clinical and Laboratory Standards Institute (CLSI) document M27-A3 [12] and using the VITEK® 2 system with the AST YS01 card; both tests confirmed the identification. *Candida tropicalis* isolates showed resistance to fluconazole and voriconazole, for which the minimal inhibitory concentrations (MIC) were 64 and 16 µg/mL, respectively; the MICs for anidulafungin, caspofungin, micafungin, and amphotericin B were 0.125, 0.03, 0.125, and 0.25 µg/mL, respectively.

The patient was diagnosed with *C. tropicalis* esophagitis. Treatment with amphotericin B deoxycholate and itraconazole with a maintenance dose was instituted. After 12 days of hospitalization, the patient developed sepsis from a pulmonary focus and his respiratory status worsened, leading to nasal intermittent mandatory ventilation. Meropenem and vancomycin were started, and itraconazole (400 mg/day) was maintained. Laboratory tests showed significant pancytopenia. Transthoracic echocardiogram revealed pericardial thickening, a sequelae of tuberculous pericarditis (Fig. 1d–f).

Two days after the end of antibiotic therapy, the patient developed new fever peaks, and the medical staff chose to prolong the medication for another 10 days. Simultaneously, herpetic lesions in the genital region appeared, and acyclovir (750 mg/d) was initiated. A modified ART regimen (tenofovir + lamivudine + dolutegravir) and tuberculostatic (COXCIP-4) therapy was given. The patient improved clinically and was stable for discharge after 30 days of hospitalization with appropriate clinical follow-up. After discharge, the patient was followed in the outpatient infectious disease service from the Hospital of Clinics at the Federal University of Pernambuco with good clinical status.

## Discussion and conclusion

We report a case of disseminated TB and fungal infection associated with complications of HIV infection. Yeast infections in PLHIV are responsible for severe clinical manifestations [10]. Oropharyngeal candidiasis is a common presenting manifestation of HIV infection, and it often develops in HIV-infected patients when the CD4+ T lymphocyte count decreases to > 350 CFU/mL. In HIV patients with CD4 cell counts of ≤ 200 CFU/mL, canker sores spread into the esophagus, transforming oral candidiasis into esophageal candidiasis [3].

Previous studies have shown that ART decreases the incidence of all HIV-related opportunistic infections, including fungal infection; however, no protective effects of such therapy on *Candida* colonization have been observed [13–15]. ART has been associated with adverse effects in the oral mucosa, including dry mouth, hyperpigmentation, and aphthous ulcers, all of which could reduce the protective effects of this yeast on the mucosa, although these findings are controversial [13].

Recurrent fungal infections have been associated with emphysematous changes and bronchiectasis in patients with HIV infection [4]. TB is a systemic disease that may involve any location and is one of the most prevalent opportunistic infections in PLHIV [6, 7, 16]. Pericarditis is related to pathologies associated with AIDS. In Africa, TB is responsible for 100% of pericarditis cases in individuals with this syndrome [5, 17, 18]. In Brazil, tuberculous pericarditis plays an essential role among HIV pericardial effusions and is usually associated with several opportunistic agents [5, 18].

The patient had esophagitis candidiasis associated with tuberculous pericarditis in the case described. However, he progressed well and was discharged. Fungal infections continue to contribute significantly to HIV-related mortality. Early and accurate diagnostic measures considerably increase therapeutic success [19].

It is essential to publish cases of TB associated with mycoses in HIV-positive patients because knowledge of the true incidence contributes to formulating public policies to prevent and control these infections and facilitate early diagnosis and proper treatment.

## Data Availability

The authors agree to make the images and data described in the manuscript freely available.

## Abbreviations

AIDS: Acquired immunodeficiency syndrome
ART: Antiretroviral therapy
CD: Cluster of differentiation
COXCIP-4: Rifampicin, isoniazid, pyrazinamide, and ethambutol
HIV: Human immunodeficiency virus
PLHIV: People living with HIV
UNAIDS: Joint United Nations Programme on HIV/AIDS
